# Failures in reproductive health policy: overcoming the consequences and causes of inaction

**DOI:** 10.1093/pubmed/fdy131

**Published:** 2018-08-18

**Authors:** Jonathan Sher, John W Frank, Lawrence Doi, Linda de Caestecker

**Affiliations:** 1 Independent Consultant on Preconception Health, Edinburgh, UK; 2 Scottish Collaboration for Public Health Research and Policy, Usher Institute of Population Health Sciences and Informatics, University of Edinburgh, Edinburgh, UK; 3 Director of Public Health, NHS Greater Glasgow and Clyde, Glasgow, UK

**Keywords:** evidence-based policy, fetal alcohol spectrum disorders (FASD), folic acid, preconception health, (public) health policy, pregnancy, prevention of birth defects, reproductive health, valproate

## Abstract

It is assumed that long-established research findings and internationally accepted evidence should, and will, be translated into policy and practice. Knowledge about what prevents harm and promotes health has, in fact, guided and resulted in numerous beneficial public health actions. However, such is not always the case. The authors examine three notable, and unwelcome, exceptions in the UK—all in the field of reproductive health and all focused on the period prior to pregnancy. The three examples of counterproductive inaction discussed are: fortifying flour with Vitamin B9 (folic acid); preventing foetal alcohol spectrum disorders; and reducing risks and better regulating a highly teratogenic medication (valproate). The adverse consequences, as well as the causes, of inaction are analysed for each example. Reasons for optimism, and recommendations for overcoming inaction, are also offered, in particular, greater priority should be accorded to preconception health, education and care.

Public health has a long and generally proud history of protecting populations from serious harm, preventing predictable dangers, minimizing unreasonable risks and intervening to solve major collective crises.^1^ Some public health measures have been instigated under extraordinary pressures—e.g. the rapid spread of little-known diseases—on the basis of educated guesswork and/or applying the Precautionary Principle, rather than scientific certainty.^2–5^

Sometimes, the choice has been made not to act at all. The reasons for not acting range from well-founded concerns about violating the fundamental principle ‘first, do no harm’ to the presence of genuinely contradictory evidence in relation to the proposed measure. Under such circumstances, restraint is usually the better part of wisdom.^6^

In contrast, the focus of this article is on public health actions that should have been implemented in Scotland (and the rest of the UK) years ago, but were not. In this overview, we explore the drivers of inaction within our society, and especially by our public bodies, when extensive scientific evidence has long justified preventive policies that have not been pursued.

The three illustrations of such inaction considered here are:

- Fortifying flour with vitamin B^9^ (folic acid);
- preventing fetal alcohol spectrum disorders (FASD); and
- valproate risk reduction and prescribing regulations.

Each of these examples has a unique history, but they share three characteristics. First, international evidence favouring these public health actions is long-standing and rock-solid; second, they all speak to the rights, empowerment and wellbeing of women of childbearing potential (and to a lesser extent, prospective fathers); and, third, the adverse impacts of inaction are much more frequent and more profound than acknowledged.

## Profiles in procrastination

### Case 1: Not fortifying flour with vitamin B^9^

Ironies abound in this example. A total of 80 countries around the world mandate the fortification of flour, or another staple grain, with vitamin B^9^ (folic acid)—but not the UK.^7^ This is true even though the British Medical Research Council funded the still-lauded, 1991 randomized control trial that provided the scientific foundation for international fortification.^8^ Following 2 decades of closely monitored implementation, no country has ever had cause to discontinue mandatory vitamin B^9^ food fortification.

The justification for significantly increasing women’s blood folate levels prior to conception is beyond scientific doubt. An adequate level of folate (primarily from folic acid) prevents more than two-thirds of neural tube defects (NTDs). But, the preventative benefit is achieved only if that higher level is reached during the months before conception and at least the first month of pregnancy (when the neural tube forms).^9^ NTDs include not only spina bifida and four forms of disordered brain development among live births, but also lead to miscarriages, voluntary terminations, stillbirths and infant deaths.

Only a minority of prospective mothers have adequate folate levels at the start of their pregnancy—almost always the result of an extended, daily, individual regimen of vitamin B^9^ (folic acid) supplementation.^10^ Among women of childbearing potential, the prevention benefits are realized less frequently by those with low socioeconomic status. This inequality is exacerbated by the fact that roughly half of all UK pregnancies are unintended or mistimed. Twenty years of actively promoting ‘voluntary supplementation’ has not succeeded.^11^

For the sake of both primary prevention and reducing inequalities, the solution is to mandate vitamin B^9^ fortification of all wheat (but not wholegrain) flour and/or other grains. This still allows consumers to choose non-fortified products and voluntary supplementation, if they prefer.

The lead researcher for the original 1991 RCT is Dr Nicholas Wald, later honoured with a knighthood for his contributions to global public health. And yet, Professor Sir Nicholas’ advice to mandate fortification of flour with vitamin B^9^—as a matter of urgency—has been ignored for more than 25 years within the UK.^12^

Major new research from Wald *et al*.^13^ removes the last medical-minority doubt about folic acid fortification. It reveals that fears of an ‘overdose’ are groundless, as there are no ill effects from ingesting any plausible level of vitamin B^9^—bodies merely eliminate any excess.

Repeatedly, and in great detail, the UK’s Food Standards Agency’s own Scientific Advisory Council on Nutrition (SACN) has examined the evidence on the pros and cons of fortification. It also assessed the evidence from all the countries mandating fortification. Each time, SACN has publicly endorsed adding vitamin B^9^ to flour without reservation—but also without success.^14^

The final irony is that the UK has already mandated and implemented the fortification of (non-wholegrain) wheat flour with four health-promoting ingredients—including both vitamin B^1^ (thiamine) and B^3^ (niacin)—for more than half a century.^15^

### Case 2: Minimizing the existence and importance of foetal alcohol harm

Starting with the 18th century Gin Act, UK governments and campaigners have tried to reduce alcohol harm. However, this general aspiration rarely extended to discouraging alcohol consumption during pregnancy. In fact, alcohol was used to offset some gestational symptoms.^16,17^

The term ‘Fetal Alcohol Syndrome’ first appeared in ‘The Lancet’ in 1973.^18^ Following up in Scotland, Dr Forrester Cockburn, Professor of Child Health at Glasgow University/Yorkhill Hospital, co-authored a 1983 article in the ‘British Medical Journal’ on the dozens of FAS cases quickly confirmed locally.^19^ The entire topic then virtually disappeared from the medical literature and health policy for more than 2 decades in Scotland/UK.^20^

Over the next 35 years, full-blown FAS came to be understood as constituting only ~10% of cases of the much broader entity, FASD.^21^ There is longstanding scientific agreement that alcohol is a teratogenic agent, passing easily through the placenta, un-metabolized by the foetus. Among those affected, the harm typically includes irreversible neurodevelopmental, cognitive and behavioural impairment.^22,23^

Amidst recent attention to ‘Scotland’s unhealthy relationship with alcohol’—including landmark minimum unit pricing legislation implemented in Scotland in 2018—FASD is mentioned only in passing.^24,25^ This FASD policy vacuum is especially noteworthy given that women in Scotland and the rest of the UK are equal consumers of alcohol in the heaviest-drinking World Health Organization region.^26^ FASD remains a cultural, professional and governmental ‘blind spot’ allowing foetal alcohol harm and its lifelong consequences to continue unabated. Even minimum unit pricing may not solve the problem.^27^

### Case 3: Failing to control access to, and gain informed consent about, valproate prescribing for women of reproductive age

French scientists serendipitously discovered in the early 1960s that valproate had anti-seizure properties. The US Food and Drug Administration (FDA) first approved it as an epilepsy medication 40 years ago (1978). The reality was confirmed in the 1980s that valproate was an especially powerful teratogen—with up to 40% of exposures during pregnancy resulting in significant physical and neurodevelopmental birth defects.^28^ Thus, the basis for informing, counselling and controlling access to valproate among women of childbearing potential has been known for at least 25 years.^29^

Across the UK, many women having epilepsy and prescribed valproate have received ‘preconception’ counselling (including switching medications pre-pregnancy). Similarly, no competent doctor would ‘start’ prescribing valproate to a visibly pregnant woman. There are automatic warnings and contraindications built into the prescribing system.

Recently, a widespread, clear warning has been issued across the UK against prescribing valproate, for any reason, to women of childbearing potential.^30^ And yet, in recent years, 92 000 valproate prescriptions annually have been given to young girls and women of childbearing age, in England alone.^31^

Recent public outcry in France has now reached the European Medicines Agency (EMA).^31,32^ The EMA held its first-ever Public Hearing in September 2017 to address problems and possible solutions related to valproate.^33^ In 2018, the EMA, and subsequently the UK Government and Irish legislature, finally recommended significantly stronger measures to reduce exposure to valproate during pregnancy (ranging from clearer warnings to better informed consent procedures) and prior to conception (e.g. risk assessments, counselling and contraception as needed, while taking valproate).^34–36^

These are welcome steps forward, but they beg the question: ‘Why were not such sensible public health measures implemented over the past 3 decades, since the requisite scientific knowledge was already firmly in place?’^37^

## The price of passivity

It is one thing to refrain from initiating public health and clinical actions when credible evidence and understanding are sparse or contradictory. But, it is quite another to have had so much certainty for so long, and yet fail to act.

Major and enduring damage was done in the UK over the past decades to the thousands of people (both living and dead) who have been affected by preventable NTDs, FASD and exposure to valproate *in utero*.^38–40^ There is no cure for spina bifida, anencephaly or other NTDs; no one ‘outgrows’ foetal alcohol harm; and, none of the valproate-induced birth defects are fully rectifiable.

Adding insult to injury, ‘secondary effects’ associated with each of these birth defects compromise lives and life chances.^41^ For instance, victims can experience stigmatization, bullying, or exclusion from school. Their parents, siblings, family members and carers have also had their work, personal and economic lives radically changed by their responsibilities toward those with FASD, NTDs or foetal valproate harm. Adverse pregnancy outcomes (e.g. miscarriages) also come with a significant human cost.

The high financial/economic burdens associated with NTDs, FASD and valproate-harm have been formally but incompletely estimated—e.g. the familiar reference to ‘million dollar FASD babies’ in Canada.^42^ Beyond these tangible burdens on the public purse and the human costs noted above, there is a broader professional and societal price tag for inaction, including loss of confidence in public health policymakers and practitioners, and questioning their commitment to reproductive health and primary prevention.

## The causes of inaction

We suggest that prolonged inaction on these pregnancy-related public health measures can best be explained by cultural factors in Scotland and the rest of the UK. ‘Cultural’ in this context refers to the ‘prevailing’ beliefs, values, assumptions, social norms, traditions, language, attitudes and socialization processes that shape collective thinking, reactions and actions.

‘First, the UK’s public policy apparatus operates in a fundamentally reactive, crisis-driven manner—and FASD, NTDs and valproate misuse have not been perceived by the “powers that be” as crises.’ A hallmark of the current era is a recurring series of inquiries to apologize for, and ‘learn lessons’ from, numerous crises—from child sexual abuse to the Grenfell Tower fire—that were predicted, but not prevented. The UK’s cultural definition of ‘heroism’ and ‘leadership’ is deeply rooted in the idea of taking bold actions to deal with an out-of-control crisis.^43^

There is plenty of rhetorical support in favour of primary prevention and preventative spending, but that is neither honoured nor reflected in the actual distribution of human, institutional and financial resources and rewards. Scotland’s respected 2011 Christie Commission on the Future Delivery of Public Services concluded that 40% of all public expenditures were driven by ‘failure demand’, i.e. reacting after the fact when prevention would have been wiser and less expensive.^44^ Due to the combination of austerity and recession—two crisis-provoking events—that percentage has subsequently risen.^45^

‘Second, there is an abiding cultural discomfort with sexual/reproductive matters; the UK has not fully disentangled itself from a global tendency of not prioritizing things related to pregnancy/parenthood—perhaps because they are considered as “women’s business”.’^46^ This reflects intrinsic sexism and the remnants of a patriarchal society (e.g. the inclination to blame women, rather than empower and support them in relation to their own reproductive goals/lives).

It is neither inevitable, nor a coincidence, that roughly 50% of UK pregnancies are unintended or mistimed—and that nearly one in six pregnancies are terminated (higher in England than Scotland).^47^ Most UK babies are conceived in the context of ambivalence about, and/or lack of preparation for, parenthood. This means fundamental questions frequently go both unasked and unanswered. As a culture, the UK exhibits an extraordinarily ‘laissez faire’ attitude toward preparing and supporting the next generation of mothers and fathers.

‘Third, our three illustrations shine different lights on how language can undermine desirable outcomes.’ Wording matters in the flour ‘fortification’ debate. ‘Folic acid’ may not have been as good a choice for reassuring policymakers and the public as ‘vitamin B^9^’. Adding a vitamin to our food sounds more benign than adding an acid. That may help explain why ‘niacin’ (vitamin B^3^)—rather than the more ominous-sounding chemical name for it, ‘nicotinic acid’—has been added uncontroversially to UK baked goods for half a century.

Valproate also offers a lesson about terminology. Current attempts to alert the public about significant, proven risks of taking valproate during pregnancy often forget that women of childbearing potential are rarely prescribed generic ‘valproate’. Instead, women in Europe perceive themselves as taking one of the 30+ brand-name versions of valproate (from Absenor to Hexaquin). For many people, it is challenging to remember brand names, let alone the active ingredient(s). This represents a crucial and dangerous linguistic disconnect.

‘Fourth, the UK’s relatively non-litigious culture—while a blessing in many respects—may have removed one powerful motivation for implementing these three public health measures.’ Samuel Johnson’s famous 18th century observation—‘Depend upon it, sir, when a man knows he is to be hanged in a fortnight, it concentrates his mind wonderfully.’—may be applicable here today. If the chances of being sued, charged with an offence, publicly shamed or held accountable in other meaningful ways for failing to act are minimal, then the impetus to implement is significantly diminished.

‘Fifth, there continues to be ‘wilful ignorance’ across Scotland and the rest of the UK about the prevalence of FASD, NTD-affected pregnancies and valproate-related birth defects.’ The absence of Scottish/UK epidemiological evidence—and other data about the consequences/costs—of all three problems is a choice, not an inevitability. Ignorance has not been ‘bliss’ for those harmed by this choice.^48^

## Replacing inaction with accomplishment

If cultural factors best explain why these three public health measures should have been implemented—but have not—then focusing on the cultural side seems the most likely way to achieve a different, better result.

We see several reasons for optimism.

- Citizens’/victims groups have very recently persuaded both the mass media and politicians to take seriously the prevention of valproate exposure during pregnancy. Some of the language from top politicians suggests an underlying cultural shift. The UK’s Health Secretary in February 2018 told the Westminster Parliament that: ‘We must acknowledge that the response to these issues from those in positions of authority has not always been good enough. Sometimes the reaction has felt too focused on defending the status quo.’^49^
- Scotland’s Chief Medical Officer has been championing the concept of ‘Realistic Medicine’ with widespread national/international agreement and a growing number of examples in practice.^50^ This is explicitly a cultural shift based upon six principles, including: ‘shared decision-making’; a ‘whole person approach’; and, ‘better managing risks’. The CMO notes: ‘… just as opinion coalesced around the advent of professionalism in the 19th century, there exists a broad international movement… to co-create health between practitioners and citizens as an inviolable standard.’^51^
- Scotland’s Minister of Public Health launched an exploration of using devolved powers to ‘go it alone’, if the UK continues to refuse mandatory vitamin B^9^ fortification. Acknowledging that UK-wide legislation is best, this Minister (and her Welsh counterpart) jointly urged the UK Health Secretary to act in late 2017 and again in 2018.^52,53^ An earlier Member’s Bill to fortify flour was passed by the House of Lords, but disappeared when the Prime Minister dissolved Parliament for a snap election.^54^ A 2018 Westminster debate may revive its prospects.^55^
- Particularly in Scotland, modest steps have been taken, and more are underway to prevent, identify and treat FASD. These range from the only online NHS course/resource for all professionals on foetal alcohol harm—through the planned 2018 publication on FASD clinical practice from the Scottish Intercollegiate Guidelines Network (SIGN)—to the first recognition of FASD in statutory guidance since Scotland enacted the Additional Support for Learning Act 2004.^56,57^
- There are Scottish/UK models of building broad coalitions to resolve other key public health issues. These brought together government, voluntary, academic, media and private sectors, as well as individual champions in effective and sustained ways. These successful precedents achieved major changes in culture, law, policy and practice—such as reducing drink driving, the smoking ban and violence reduction (especially knife crime)—and should encourage and inform similar public health efforts around NTDs, FASD and valproate exposure.
- The relevant precedents are not limited to public health, as there is a great deal to learn, and adapt, from other profound social/cultural movements that sparked change. These include lessons from the history of science and medicine, but especially from the women’s rights movement and the children’s rights movement. The whole ‘rights’ ethos provides a useful foundation for building both an individual sense of agency and a collective sense of responsibility to prevent harm and promote wellbeing/equity.

## Prioritizing preconception health

The biggest still-missing element in overcoming longstanding inaction on these three public health measures is a movement in favour of—and culture changes that give priority to—preconception health, education and care across the life course.^58–60^ There is an antenatal component to each of the three examples, but they all require much more attention to what happens (or fails to happen) prior to pregnancy.

That, in turn, requires a cultural change beyond the traditional binary choice between either avoiding pregnancy or being pregnant. There are two additional, often-overlooked stages between ‘not pregnant’ and ‘pregnant’. One is being ready, willing and able to make empowered, informed choices about whether and when to become a parent. The other is preparing for pregnancy.^61^ A society that takes preconception (before the first pregnancy) and interconception (before the next pregnancy) seriously is one in which inaction on NTDs, FASD and valproate would not be tolerated or perpetuated.^62^

Achieving safer pregnancies and thriving babies is within reach here and now. The key is finally taking robust action on these public health measures. The next generation deserves no less.
